# Supplementary material for: Temporal SNR optimization through RF coil combination in fMRI: The more, the better?
Source: PLoS One. 2021 Nov 8;16(11):e0259592. doi: 10.1371/journal.pone.0259592 (PMC8575292; doi:10.1371/journal.pone.0259592)
Supplement: S1 File — (PDF) [file pone.0259592.s001.pdf]

STARC algorithm

Initialization :

epi_uncombined ← reconstructed uncombined fMRI data (dimensions are [Cha, Rep, Lin, Col, Par])

SOS_mean ← the temporal mean image from epi_uncombined with sum of square

//this loop can be parallelized

Loop: each voxel across spatial dimensions (Col, Lin and Par)

A ← matrix containing signals time courses from all 32 channels (32 columns) for the current voxel

u ← temporal mean of A

b ← SOS_mean of the current voxel

X ← $\frac{b\,cov(A)^{-1}u}{u'cov(A)^{-1}u}$

STARC_reco ← AX *//result of the STARC optimization for the current voxel*

End of loop

STARC$_{tsm}$ algorithm

Initialization :

epi_uncombined ← reconstructed uncombined fMRI data (dimensions are [Cha, Rep, Lin, Col, Par])

SOS_mean ← the temporal mean image from epi_uncombined with sum of square

STARC_tsm_reco ← zero matrix of dimensions [Rep, Lin, Col, Par]

D ← design matrix of the paradigm of dimension [Rep, NbVariables]

// D must contain a constant column to estimate the temporal mean

D^+ ← $(D'D)^{-1}D$ *//pseudo inverse of D*

P ← Id-DD^+ *//Id is the identity matrix*

c ← contrast vector selecting the mean estimator

//this loop can be parallelized

Loop: each voxel across spatial dimensions (Col, Lin and Par)

A ← matrix containing signals time courses from all 32 channels (32 columns) for the current voxel

u ← $c'D^+A$

b ← SOS_mean of the current voxel

X ← $\frac{b\,cov(PA)^{-1}u}{u'cov(PA)^{-1}u}$

STARC_tsm_reco← AX *// result of the STARC$_{tsm}$ optimization for the current voxel*

End of loop
