# Supplementary material for: Temporal SNR optimization through RF coil combination in fMRI: The more, the better?
Source: PLoS One. 2021 Nov 8;16(11):e0259592. doi: 10.1371/journal.pone.0259592 (PMC8575292; doi:10.1371/journal.pone.0259592)
Supplement: Attachment — Submitted filename: ResponseToReviewers.docx [file pone.0259592.s002.docx]

**Reviewer #1:** The authors have presented an investigation into the impact of several RF receive array coil combination strategies on temporal signal-to-noise ratio (tSNR) and functional MRI detection sensitivity. This included an analysis of a previously proposed voxel-wise, data-driven tSNR-weighted combination, termed STARC, and variants of this technique that the authors proposed based on a pre-scan estimation of the STARC coil combination weightings and a related method based on the t-score of the mean. The authors found that, despite large improvements in tSNR, most of the STARC techniques had no benefit or and even reduced the detection sensitivity to their stimulus.

Very often, tSNR is the primary metric for assessing new methods in fMRI, but the authors argue here that optimizing tSNR is not necessarily entirely reflective of fMRI detection sensitivity.

**We agree with the reviewer. However here we want to stress that this result seems to be a consequence of a data-driven optimization. Our results do not contradict this principle with more standard approaches like cov-SOS, where the higher the tSNR, the better.**

I think this is a valid point, although I am not convinced based on the experiments and analyses performed here that the authors’ conclusions are entirely accurate. I think that with additional experimental data, these conclusions could be much more well-validated and that with alternative, more common, approaches to analyzing the same data, the results may change significantly. I don’t think acquiring new data is necessary for the manuscript, but it could significantly increase its impact. Without new data, the conclusions will need to be significantly tempered down in terms of scope of applicability. For these reasons, I suggest a major revision for this manuscript.

**We agree with the reviewer. We have extended our discussion to emphasize that our observations apply only to this regime. However, the other referee pointed out a reference to us indicating some results agreeing with our observations at 0.8 mm isotropic resolution. So we have elaborated the discussion and caution the reader that different results can be obtained depending on the nature of the noise.**

Major comments:

1. The authors have not characterized the noise regime, physiological or thermal noise dominated, that their protocol is operating in. Given that the focus of the paper is testing alternative coil combination strategies that can potentially reduce physiological noise and temporal instability, this is a significant omission. In Fig. 1, the covSoS tSNR values are in the range of ~0–70, this would appear to be thermal noise dominated. Many of the conclusions in the manuscript are quite strong although it seems like the conclusions could differ if the data were in a different noise regime and not just acquired under the single protocol used in this study. Ideally, one would like to see the impact of the different coil combinations under a range of SNR conditions (e.g., voxel size, flip angle, echo-time variation) as is commonly done in similar papers, e.g., refs 6, 7, 9, 15. In lieu of acquiring new data under varying protocols, can the authors elaborate on how the noise regime might impact the different STARC coil combinations compared to covSoS and reframe their conclusions in terms of their particular acquisition?

**As indicated by the referee, the tSNR of the voxels spans the range 0-70 which means some voxels are in the thermal noise dominated regime and some are at the border of thermal and physiological noise regimes. What we have tried to convey is that any redundancy of signal seen by different channels can be cancelled to some extent by the algorithm. We now provide some plots that show that some voxels are in the thermal and physiological noise regimes, depending on the receive channel. We specify that our conclusions are made for data-driven optimizations for a HCP based fMRI protocol.**

2. To calculate tSNR for the coil combination and for the analysis of the coil-combined data, the authors have taken the straightforward ratio of the signal mean over the standard deviation in their task fMRI data. A more appropriate and common calculation of tSNR in task data is based on normalizing by the standard deviation of the residuals of the GLM, as is described, for example, in Murphy et al. (ref. 14) and Corbin et al. (ref. 11). When the desired signal fluctuations are properly modelled in the GLM, this should ensure that they are not included as noise. Ideally, this may then provide the same coil weightings as the STARC_ps method. The authors should consider combining their data using this more common tSNR calculation. Also, since the GLM would have to be run twice, it would make sense to consider possibly inflated statistics, like what was performed for STARC_tsm.

**Thank you. This is exactly what was attempted by optimizing the t-score for the mean (STARC_tsm_). The double dipping investigation is precisely the inflated statistics the referee mentions. We have tried to clarify this aspect.**

3. The authors claim in the Discussion that the STARC method penalizes high-intensity voxels and promotes weak-intensity voxels (pg. 12, lines 324–329). This seems to be the meat of the problem (and likely related to my comment above) and it is surprising that this is only mentioned in the Discussion. While this may be intuitive for the authors, it would be great to see this explored more quantitatively in the Results with some concrete examples. Is this apparent in the resulting time series? This is in…stark contrast…with the originally presented method from Huber et al., where tSNR and z-statistics both increased significantly. The authors should discuss why the discrepancy may exist.

**Thank you. We have incorporated two new figures that show the coil combinations with respect to signal strength in addition to a time series example of two voxels. Our understanding is that stronger signals can lead to more variance (including neural activations) and therefore are penalized. This can also be seen in the analytical derivation of STARC we report. Consider the time series covariance matrix** $\boldsymbol{\Psi}_{\boldsymbol{t}}$**. For simplicity, we shall assume it is diagonal. Reconstructing then by taking the inverse of this matrix will penalize the channels that see the more variance. Unfortunately, part of this variance arises from activations. We have tried to emphasize this aspect in the revision of the manuscript.**

**We can hardly reconcile our results with Huber’s because we do not have access to the data. We would like to point out however the abstract mentioned by the second referee which agrees with our observations as well. Despite yielding higher tSNRs, STARC returned lower t-scores at 9.4T in a laminar fMRI study (0.8 mm isotropic resolution). One possibility for the mentioned discrepancy with Huber’s results is that the latter employed a numerical algorithm to optimize with constraints on the weights. This may have indirectly acted as « damage control ». Our approach did not have such constraints and incorporated an exact analytical solution. We did not include such constraints by choice to emphasize the risks of searching for ever higher tSNRs. Another possibility is that the particular scan may have exhibited large scanner instabilities, that STARC may have helped compensating. This is now elaborated in the discussion.**

4. pg. 6, line 132: It’s not entirely clear to me how the authors arrived at the solution for the coil combination weighting matrix, X = –1/2 cov(A)^-1 u. This appears as if the authors arbitrarily set the Lagrange multiplier lambda to 1. I would expect the solution to have the parameter b in it. Considering that b is also somewhat of an arbitrary parameter, maybe this explains the solution, however, it was previously stated that b was constrained to be equal to the sum-of-squares mean value.

**Thank you. Lambda was missing in the expression. Since X is a weighting vector, it is possible to scale the solution in order to match the constraint by arbitrarily setting the Lagrangian multiplier with no impact on tSNR.**

Minor comments

5. In STARC_tsm, my understanding is the GLM is run voxel-wise and per receive channel to estimate the t-score on the mean-based weights. Can the authors explain if they applied typical preprocessing steps (e.g., motion correction, detrending, smoothing, etc.) to every channel’s image and repetition prior to coil combination and why or why not?

**Sorry for the confusion. All coil combinations were performed before any pre-processing step to maintain a more practical workflow. In STARC_tsm_, we directly develop an expression of the t-score that depends on X. The optimization yields then the optimal coil combination that will have the highest t-score. We add in the expression the right contrast vector c to select the estimator of the mean (hence the name “t-score for the mean”). That way, we optimize a coil combination for the t-score for the mean (i.e. the temporal mean normalized by the standard deviation of the residual of the GLM).**

6. Can the authors please provide some more details on the pre-scan acquisition for the STARC_ps_ method? How many repetitions were acquired? Presumably, unlike the calculation of the thermal noise covariance matrix, RF excitation was enabled for this scan?

**Thank you. The RF excitation indeed was enabled to attempt obtaining signal and variance related to systematic scanner instabilities and physiological noise (total noise covariance of the time-series). The pre-scan was performed with the same sequence and parameters as the fMRI scans but with 100 repetitions. The weights were computed using the STARC algorithm on the pre-scan but were applied to the fMRI scans. We have clarified this in the methods section.**

7. I think the subtitle of the manuscript, “How high can we go?” is not totally appropriate for this study since I don’t think the question was really answered. I would reconsider the subtitle for something that better reflects the experiments performed and the conclusions drawn.

**Thank you. The referee is correct. We would suggest replacing the statement by « the more, the better? »**

8. pg. 4, line 79: The reference number for Triantafyllou et al. should be [15]

**OK.**

9. pg. 5, line 128-129: Please consider rephrasing, “The Lagrangian multiplier method for the yields for the Lagrangian”

**OK.**

10. pg. 6, line 154: I believe “projector” should be “projection”?

**The referee is right.**

11. pg. 6, line 157: The final ‘c’ in the denominator should not be transposed.

**The final comma at the end of the expression was confused with the transpose sign; we added some space for clarity.**

12. pg. 11, line 295: Should be figure caption ‘b’, not ‘c’

**OK.**

13. pg. 12, line 324: seems like a word or two is missing from “noise is reduced but activation spikes too”

**Thank you. It has been corrected.**

**Reviewer #2:** Summary

Jamil et al. investigate the effect of different data-driven coil combinations methods that either maximise tSNR or the t-score of the mean on the t-score of the activation and the activation cluster size. They show that contrary to when the idea was first proposed (Huber et al., 2017), STARC or any of its variants do not increase t-scores for activation, which is in line with previous, initial pilot experiments (Kashyap et al., 2018).

**Thank you for pointing this interesting reference that agrees with our observations. Perhaps one contribution of our work here was to provide an analytical solution for the STARC and STARC_tsm problems.**

This study addresses an interesting topic, which has been discussed in the community, but not thoroughly investigated so far. In addition, including the effect of double-dipping here provides a valuable reference given the current popularity of data-driven denoising strategies, and I recommend the authors to at least make this part of their code available.

**Thank you. We now include in the supplementary material the pseudo-code that performs t-score for the mean optimization.**

I have a few questions regarding the interpretation of the results and the effect of physiological noise (below). Overall, this study provides a valuable addition to the literature.

Major points

1. Line 254ff and 329ff: You mention in the results and the discussion that the optimization strategy is highly scan-dependent. This is quite a surprising result. Do you have any more insights into why this is the case? Given the relatively smooth coil sensitivities, and if the location of physiological noise sources does not change drastically from one scan to the next, it seems that the driving factor behind this is still unclear. Maybe comparing the coil combination weights for these two scenarios would provide some hints?

**For the prescan approach, our understanding is that a relatively small sample of noisy signal can yield fluctuations of the noise covariance matrix. Take for instance in Matlab A=randn(100,32). Then Cov(A) will not strictly be the identity matrix despite having in principle perfectly uncorrelated and equal variance data. Repeating this operation many times will show fluctuations of the covariance matrix. Only when the number of rows grows significantly, shall the returned covariance matrix converge towards the Identity matrix. This shows that a time-series covariance treatment for coil combination will be somewhat sensitive to the particular signal instance. This is now elaborated in the discussion.**

2. Fig 3: Given that the result cannot be fully explained and the effect seem to be somewhat elusive, could you provide more data, for example in the form of scatter plots for the other participants and task conditions in the supplementary?

**Thank you. We now provide scatter plots for auditory task conditions and also including all volunteers.**

3. Line 272ff: Similar to my questions before, can you provide examples that quantify the interscan motion and its effect on the coil combination weights?

**This is an interesting question. STARC and it variants are voxel-wise operations and are thus sensitive to interscan motion. Intuitively, the influence of interscan motion would be seen by all channels according to the sensitivity profiles so that motion-induced signal variations could potentially be compensated. However, it is hard to quantify the effect of motion on the weights as we compare a 1×32 vector (coil combination) per voxel and a matrix of 6×260 elements (6 motion parameters and 260 volumes). Moreover, knowing the exact influence of the motion on the signal would require isolating all other sources of signal variations. Without additional measurements aiming at quantifying them, the task appears impossible.**

4. Line 325ff: Can you provide examples of coil combination weights to show how strongly STARC penalizes coils with highest signal?

**Thank you. This was requested also by the other referee and we now incorporate new figures illustrating this important aspect. This behavior can be understood also by the analytical solution, as we now emphasize in the text.**

5. Line 345ff: Triantafyllou et al. (2016) actually discuss the presence of physiological noise which does not scale with signal strength. Could you comment on why this may or may not play a role for your study.

**Given the strong proportionality between signal strength and variability in our data (see fig 5.), these noise components were not the prime source of signal variability and thus probably did not have an important influence on the STARC weights. However, any noise source, whether it is proportional to signal strength or not, if it is seen simultaneously by the different channels, can be compensated to some extent by the algorithm. For instance, if we assume we only have perfectly correlated noise among the 32 channels (i.e. same noise waveform up to a scalar constant), then the algorithm can perfectly suppress it and obtain infinite tSNR. We now mention this in the discussion.**

6. In general, can you provide more details on what you include in your physiological noise definition, and its statistical properties? Given that signals from breathing or cardiac activity are noise by definition only (i.e. they cause ‘true’ changes for example in T2*), the rationale why different channels should pick up these signal differently, and in such a way that they can be avoided during coil combination while the activation signal remains untouched, isn’t fully clear.

**Thank you. The STARC approach was initially motivated by the presence of scanner instabilities, GRAPPA ghosts or phase errors, which are space and coil dependent.**

**However, no assumptions about the noise were made in this work. Starting from the definition of the standard tSNR, we simply derive a formula that returns proven optimal coil combination in the tSNR sense. The channels see redundant information when it is activations or physiological noise equally. Therefore the algorithm manages to partly cancel them. It is precisely the point that in this approach, the algorithm cannot distinguish the activations from the undesired physiological noise, thereby motivating the investigation of STARC_tsm_, which attempted filtering out the noise without touching the activations. However, as we have tried to demonstrate, this leads to double dipping and affects the statistics of the null hypothesis. We have tried to clarify this in the methods and discussion section.**

Minor Points

7. Line 45ff: Please add relevant references (see also previous point).

**Thank you.**

8. Line 54: yet -> step?

**OK.**

9. Line 74: time-correlations -> maybe temporal correlations?

**OK.**

10. Line 79: [14] -> [15]

**OK.**

11. Line 98: For clarity, could you define ‘H’ as well?

**Done. It meant Hermitian conjugate.**

12. Line 107ff: ‘by substituting to the scalar coefficient expressing the effective strength of the physiological noise a physiological noise covariance matrix’ -> ‘by substituting the scalar coefficient expressing the effective strength of the physiological noise with a physiological noise covariance matrix’

**Done.**

13. Line 237: Why for only one task?

**We now provide additional plots for auditory task.**

14. Line 252: For readability, you could briefly summarize the ideas behind STARC, STARCps, and STARCtsm again, as not everyone might read (or remember) the theory section in detail.

**Good idea. We have included this summary.**

15. Line 265: figure 1 -> Figure 1

**OK.**

16. Line 288: fig.3.b. -> Fig. 3b

**OK.**

17. Line 295: …gain. c – Scatter … -> … gain. b – Scatter …

**OK.**

18. I would also recommend to include the ISMRM abstract by Kashyap et al. into the discussion (2018), as this corroborates your findings.

**Thank you for pointing this reference to us.**

19.References

Huber, L., Jangraw, D.C., Marrett, S., Bandettini, P.A., 2017. Simple approach to improve time series fMRI stability: STAbility-weighted Rf-coil Combination (STARC), in: Proc. Intl. Soc. Mag. Reson. Med. 25.

Kashyap, S., Fritz, F.J., Harms, R.L., Huber, L., Ivanov, D., Roebroeck, A., Poser, B.A., Uludağ, K., 2018. Effect of optimised coil-combinations on high-resolution laminar fMRI at 9.4T. Presented at the Proc. Intl. Soc. Mag. Reson. Med. 26.

Triantafyllou, C., Polimeni, J.R., Keil, B., Wald, L.L., 2016. Coil-to-coil physiological noise correlations and their impact on functional MRI time-series signal-to-noise ratio. Magnetic Resonance in Medicine 76, 1708–1719. <https://doi.org/10.1002/mrm.26041>
